# Serum uromodulin is associated with impaired glucose metabolism

**DOI:** 10.1097/MD.0000000000005798

**Published:** 2017-02-03

**Authors:** Andreas Leiherer, Axel Muendlein, Christoph H. Saely, Elena Kinz, Eva M. Brandtner, Peter Fraunberger, Heinz Drexel

**Affiliations:** a Vorarlberg Institute for Vascular Investigation and Treatment (VIVIT), Feldkirch, Austria; b Private University of the Principality of Liechtenstein, Triesen, Liechtenstein; c Medical Central Laboratories, Academic Teaching Hospital Feldkirch, Feldkirch, Austria; d Department of Medicine and Cardiology, Academic Teaching Hospital Feldkirch, Feldkirch, Austria; e Drexel University College of Medicine, Philadelphia, PA, USA.

**Keywords:** coronary patients, glucose metabolism, kidney disease, renal biomarker, serum, T2DM, Tamm–Horsfall protein (THP), uromodulin

## Abstract

Uromodulin is the most abundant urine protein under physiological conditions. It has recently been described as a serum and plasma marker for kidney disease. Whether uromodulin is associated with impaired glucose metabolism is unknown.

We therefore measured serum uromodulin and glucose traits in a cohort of 529 consecutively recruited patients.

Serum uromodulin was significantly and inversely correlated with fasting plasma glucose (*r* = −0.161; *P* < 0.001), with plasma glucose 2 hours after an oral 75 g glucose challenge (*r* = −0.158; *P* = 0.001), and with HbA1c (*r* = −0.103; *P* = 0.018). A total of 146 (27.6%) of our patients had type 2 diabetes mellitus (T2DM). Analysis of covariance confirmed that T2DM was an independent determinant of serum uromodulin (F = 5.5, *P* = 0.020) after multivariate adjustment including hypertension and glomerular filtration rate. Prospectively, uromodulin was lowest in patients with T2DM at baseline, higher in initially nondiabetic subjects who developed diabetes during follow-up (FU) and highest among nondiabetic patients (147.7 ± 69.9 vs 164 ± 67 vs 179.9 ± 82.2 ng/mL, *P*trend < 0.001). Similar results were seen with respect to prediabetes (168.0 ± 81.2 vs 172.8 ± 66.3 vs 188.2 ± 74.0 ng/mL, *P* = 0.011).

We conclude that serum uromodulin is significantly associated with impaired glucose metabolism and the development of prediabetes and diabetes.

## 1 Introduction

First described by and initially named after Tamm and Horsfall in 1950,^[1]^ and redescribed 35 years later by Muchmore and Decker as an immunomodulative glycoprotein,^[2]^ uromodulin is known as the most abundant protein in human urine under physiological conditions. It is exclusively synthesized in the epithelial cells lining the thick ascending limb of Henle loop and predominantly targeted to the apical membrane and then secreted in urine. Although, little uromodulin is basolaterally targeted and released into the serum.^[3]^

In urine, uromodulin forms extracellular filaments and aggregations via self-polymerization which capture pathogenic bacteria.^[4,5]^ This protection mechanism against urinary tract infection has been first reported in 1980.^[6]^ More recently, it has also been hypothesized that uromodulin might act as kind of a guardian against kidney disease and hypertension.^[7,8]^ This was based on data demonstrating decreased excretion of uromodulin in urine of diabetic patients compared to control subjects.^[9,10]^ However other studies did not observe such an effect.^[11,12]^

Whilst most studies in the past have looked at urinary uromodulin, two recent studies have measured uromodulin in blood. Both reported an association between uromodulin in plasma or serum and kidney function and recommended its further use as renal biomarker.^[13,14]^

As kidney disease and diabetes are linked,^[15]^ the question arises whether uromodulin is also associated with diabetes. Only few studies have also looked at this question assessing uromodulin in urine, but with contradictory results.^[9–12]^ Moreover, there are at present, no data about the association between diabetes and uromodulin in blood.

Thus, in the present study, we measured uromodulin concentration in blood serum and assessed its association with glucose traits and with relevant clinical parameters in diabetic and non diabetic patients.

## 2 Methods

### 2.1 Study subjects

From September 2005 to April 2008 we consecutively enrolled 529 Caucasian patients who were referred to elective coronary angiography for the evaluation of established or suspected stable coronary artery disease (CAD). Coronary angiography was performed with the Judkin technique and the severity of stenosis was assessed by visual inspection by a team of 2 investigators who were blinded to serologic assays as described previously.^[16]^ Coronary artery stenoses with lumen narrowing ≥50% were considered significant and the extent of CAD was defined as the number of significant coronary stenoses in a given patient. Patients with acute coronary syndromes were excluded from the study. Information on conventional cardiovascular risk factors was obtained by a standardized interview.

Type 2 diabetes mellitus (T2DM) was diagnosed according to American Diabetes Association (ADA) guidelines^[17]^ and anamnestic known diabetes. Systolic and diastolic blood pressure was measured by the Riva–Rocci method under resting conditions in a sitting position at the day of hospital entry at least 5 hours after hospitalization. Hypertension was defined according to the Seventh Report of the Joint National Committee on Prevention, Detection, Evaluation, and Treatment of High Blood Pressure.^[18]^ Height and weight were recorded, and body mass index (BMI) was calculated as body weight (kg)/height (m^2^). According to world health organization criteria, BMI ≥ 30 was regarded as obesity.^[19]^ According to National Cholesterol Education Programme ATP-III criteria,^[20]^ the metabolic syndrome was diagnosed in the presence of any 3 of: waist circumference >102 cm in men and >88 cm in women, triglycerides ≥150 mg/dL (1.7 mmol/L), high density lipoprotein cholesterol <40 mg/dL (1.0 mmol/L) in men and <50 mg/dL (1.3 mmol/L) in women, blood pressure ≥130/≥85 mm Hg, or fasting glucose ≥100 mg/dL (5.6 mmol/L).

Current smoking status was applied for patients currently smoking or having quit smoking <1 year prior to the study, the alcohol consumption status in case of any consume. The present study has been approved by the Ethics Committee of the University of Innsbruck. Written informed consent was given by all participants.

### 2.2 Laboratory analyses

Venous blood samples were collected after an overnight fast of 12 hours prior to angiography and laboratory measurements were performed from fresh serum samples, as described previously.^[21]^ Serum triglycerides, total cholesterol, low-density lipoprotein cholesterol, and high-density lipoprotein cholesterol were determined on a Cobas 6000/8000 (Roche, Basel, Switzerland).

Levels of fasting plasma glucose (FPG) were measured enzymatically from venous fluoride plasma samples with the hexokinase method (Roche Basel, Switzerland) on a Hitachi 717 or 911 (Mountain View, CA). Glycosylated hemoglobin was determined as hemoglobin A1c (HbA1c) by high-performance liquid chromatography on a Menarini–Arkray KDK HA 8140 (Kyoto, Japan). Oral glucose tolerance test (OGTT) was performed after an oral 75 g glucose challenge. The estimated average glucose (eAG) value has been calculated from HbA1c according to (HbA1c × 28.7) − 46.7^[22]^ Serum insulin was measured by an enzyme immunoassay on an AIA 1200 (Tosoh, Foster City, CA). Insulin sensitivity index (ISI) was calculated according to cederholm as follows: ISI = 75,000 + (G0 − G120) × 1.15 × 180 × 0.19 × weight/120 × Gmean × log (Imean)^[23]^ and the homeostatic model assessment (HOMA) index of insulin resistance (IR) was calculated according to the formula HOMA-IR = (insulin × glucose)/22.5 as described by Matthews et al.^[24]^ For assessing the function of β cells, HOMA-β (formula: HOMA-β (%) = 20 × insulin/[glucose −3.5])^[24]^ and the eAG/FPG ratio^[25]^ were calculated. All subjects had fasting glucose concentrations above 3.5 mmol/L, thus permitting calculation of HOMA-β and insulin concentration above the detectable limit of this method of 0.8 μU/mL.

Urinary albumin excretion was expressed as the albumin/creatinine ratio (ACR). The urinary albumin concentration was determined using immunoturbidometry (Tina-quant Albumin Gen.2 Assay, Roche Diagnostics, Basel, Switzerland). As no cystatin C measurement was available for our patient samples, the glomerular filtration rate (GFR) has been estimated using serum creatinine (measured in mg/dL) according to the quadratic Mayo Clinic equation  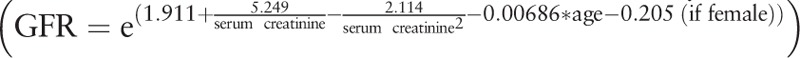


If serum creatinine was <0.8 mg/dL, 0.8 mg/dL was inserted as a value for serum creatinine. This equation gives more accurate estimates of GFR in patients with nearly normal renal function than the Modification of Diet in Renal Disease equitation^[26]^ and has been demonstrated to improve the prediction of GFR in diabetic subjects compared to other formulae.^[27,28]^ Both serum and urinary creatinine concentrations were measured using the modified Jaffe method (Creatinine Jaffe Gen.2 Assay, Roche, Basel, Switzerland). Uromodulin levels in patient serum samples were determined with a commercial uromodulin enzyme-linked immunosorbent assay kit (BioVendor, Brno, Czech Republic; catalog no. RD191163200R), specific for human uromodulin with an interassay variation less than 8%.

### 2.3 Prospective study

At the FU visit after 3.5 ± 1.1 years in our institution, a basic laboratory analysis has been performed as described above for baseline characterization and diabetes status has been assessed in 408 patients. Thirty-three patients have been deceased and 88 did not attend the FU visit by other reasons.

### 2.4 Statistical analysis

Differences in baseline characteristics were tested for statistical significance with the Chi-squared tests for categorical and Jonckheere–Terpstra tests for continuous variables, respectively. Correlation analyses were performed calculating nonparametric Spearman rank correlation coefficients. In addition, analysis of covariance models (ANCOVA) were built using a general linear model approach. For comparing the continuous or categorical variables between baseline and FU in patients, we used Wilcoxon and McNemar test, respectively. All data were analyzed according to complete-case analysis, apart from albumin creatinine ratio (ACR) in which multiple imputation was used to estimate missing data. Results are given as mean (±standard deviation) if not denoted otherwise, and *P*-values <0.05 were considered significant. Normal distribution was checked using Kolmogorov–Smirnov and Shapiro–Wilk test, respectively.

A priori sample size calculation showed that, assuming a standard deviation of 70 ng/mL for uromodulin as a continuous response variable from independent control (nondiabetic patients) and experimental subjects (diabetic patients), with 2.6 controls per experimental subject, 101 experimental subjects were needed to reject the null hypothesis that the population means of the experimental and control groups are equal with a power of 80% at an alpha-fault of 0.05. Therefore, the study (sample size: 529; 146 diabetic and 383 nondiabetic subjects) was sufficiently powered. All statistical analyses were performed with SPSS 21.0 for Windows (SPSS, Inc., Chicago, IL) and have been evaluated by an expert in the field. Power calculation was done using PS power and Sample Size Calculations 3.0.^[29]^

## 3 Results

### 3.1 Patient characteristics

Patients’ characteristics revealed a high prevalence of T2DM (25.6%), hypertension (80.9%), and current smoking (17.6%). Mean FPG was 110.7 ± 36.6 mg/dL and mean HbA1c was 6.1% ± 1.0% (43 ± 10 mmol/mol). Full study characteristics for the comparison of diabetic to nondiabetic subjects are presented in Table 1. Serum uromodulin on average was 164.7 ± 77 ng/mL (mean ± SD) with a range of 21.6–612.5 ng/mL and a median of 155.8 ng/mL. Older (>65 years) patients had significantly lower serum uromodulin concentrations than younger (≤65 years) patients (154.3 ± 69.4 vs 178.4 ± 84.4 ng/mL; *P* = 0.001) and patients with hypertension had significantly lower concentrations than those without hypertension (160.9 ± 74.0 vs 181.8 ± 87.8 ng/mL; *P* = 0.037). Men had lower serum uromodulin concentrations than women, but the difference was not significant (157.5 ± 63.3 vs 178.3 ± 96.5 ng/mL; *P* = 0.151). In addition, there was also no significant difference between obese and nonobese patients (160.9 ± 82.7 vs 166.5 ± 75.0 ng/mL; *P* = 0.272), between current smokers and nonsmokers (172.3 ± 75.6 vs 163.3 ± 77.5 ng/mL; *P* = 0.167) or between alcohol consumers and abstainers (159.2 ± 66.6 vs 166.7 ± 83.1 ng/mL; *P* = 0.664). With respect to the metabolic syndrome, affected and unaffected subjects had comparable uromodulin concentrations (163.4 ± 84.3 vs 165.5 ± 74.0 ng/mL; *P* = 0.518). There was also no significant difference between patients with and without angiographically determined significant CAD (165.4 ± 78.9 vs 164.2 ± 75.3 ng/mL; *P* = 0.934). Moreover, if we compared patients treated and untreated with acetylsalicylic acid (166.7 ± 74.3 vs 160.8 ± 83.4 ng/mL; *P* = 0.195), betablocker (162.7 ± 78.0 vs 167.5 ± 76.4 ng/mL; *P* = 0.357), angiotensin-converting enzyme blocker (165.5 ± 92.2 vs 164.6 ± 70.1 ng/mL; *P* = 0.405), angiotensin-2 antagonists (152.7 ± 60.8 vs 166.6 ± 79.2 ng/mL; *P* = 0.267), or statins (172.1 ± 84.1 vs 157.3 ± 68.6 ng/mL; *P* = 0.124) at baseline, serum uromodulin concentrations did not differ significantly.

**Table 1:**
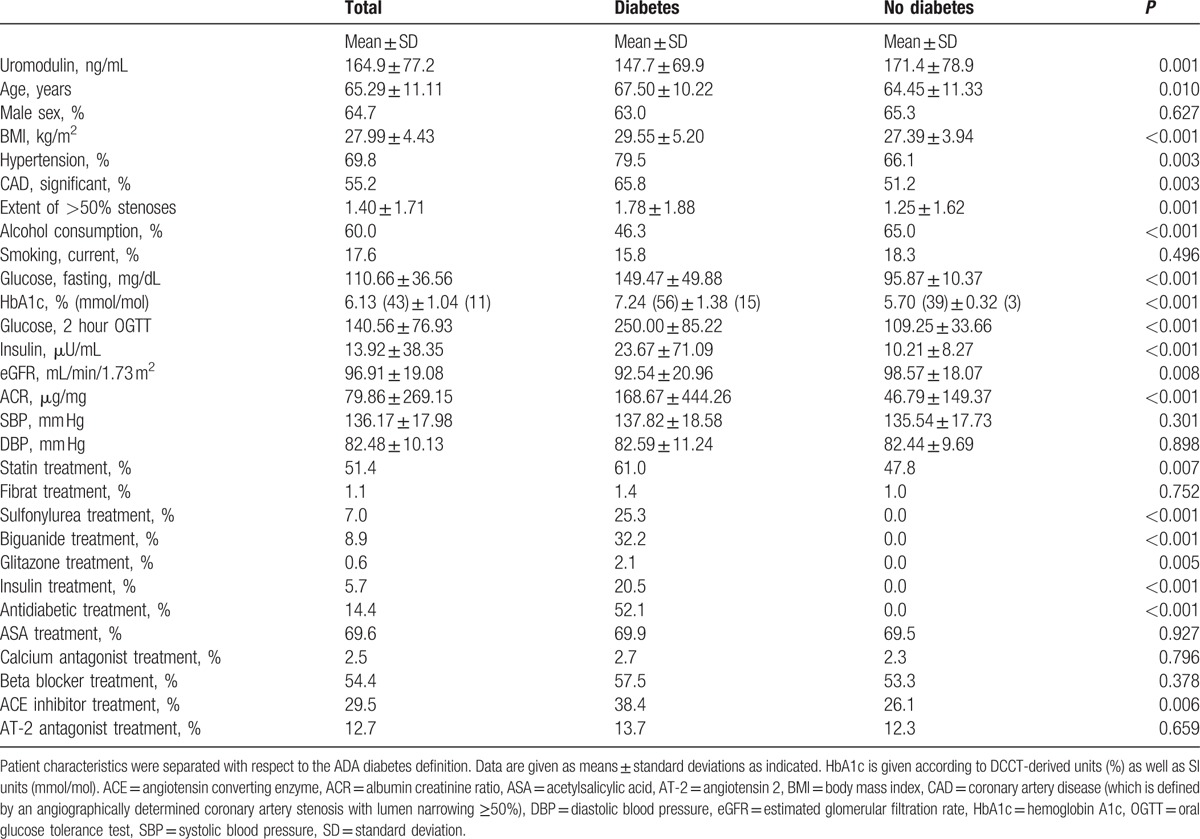
Patient characteristics.

### 3.2 Association of serum uromodulin with metabolic and glucose traits

Serum uromodulin was significantly and inversely correlated with FPG (*r* = −0.161; *P* < 0.001), with the 2 hours glucose value after a 75 g glucose challenge in OGTT (*r* = −0.158; *P* = 0.001), with mean glucose in OGTT (*r* = −0.219; *P* < 0.001), and also with HbA1c or eAG, respectively (*r* = −0.103; *P* = 0.018). Apart from that, FPG and eAG were significantly correlated (*r* = 0.637, *P* < 0.001). No correlation was seen between serum uromodulin and fasting insulin (*r* = −0.016; *P* = 0.710), 2 hours insulin in OGTT (*r* = 0.015; *P* = 0.781), or mean insulin in OGTT (*r* = 0.015; *P* = 0.781). There was also no significant correlation between serum uromodulin and the HOMA index for IR (*r* = −0.059; *P* = 0.176). With respect to diabetes duration prior to baseline, we found a slight inverse correlation with uromodulin, just failing to reach statistical significance (*r* = −0.086; *P* = 0.052).

Moreover, serum uromodulin was significantly correlated with the ISI (*r* = 0.204; *P* < 0.001). It also correlated with beta cell function according to HOMA-β (*r* = 0.096; *P* = 0.028), as well as the eAG/FPG ratio (*r* = 0.122; *P* = 0.005). In addition, serum uromodulin was also correlated with the eAG-FPG difference (*r* = .095; *P* = 0.029). Further associations are summarized in Table 2.

**Table 2:**
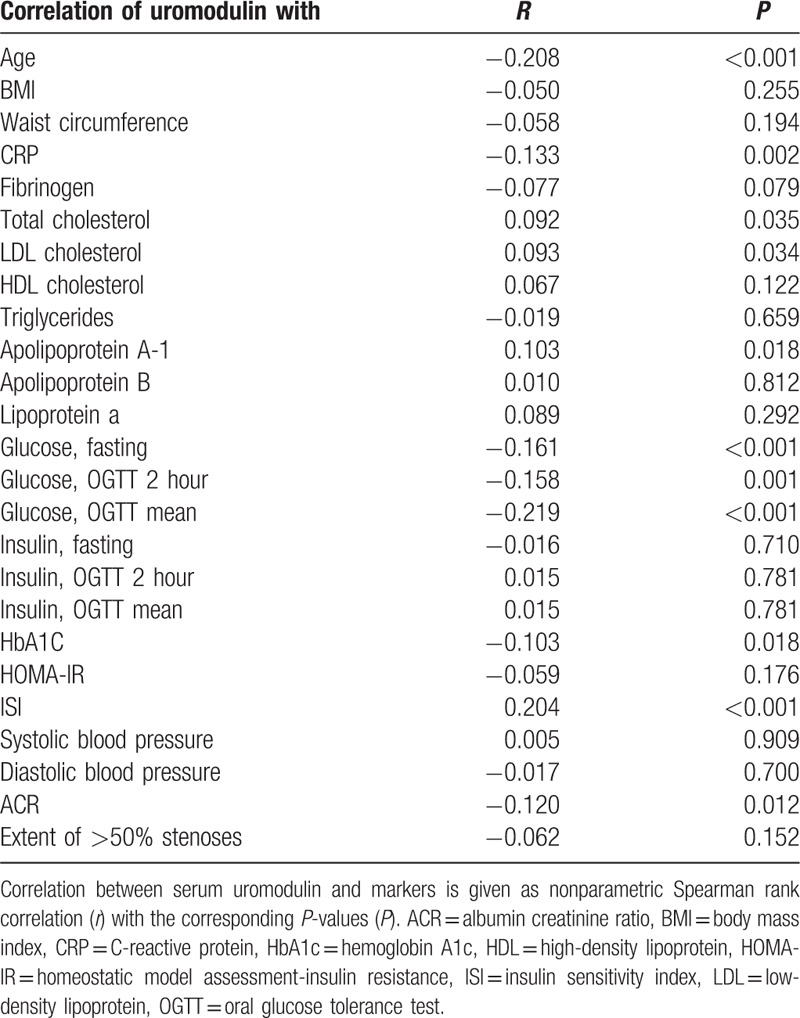
Correlation analysis between serum uromodulin and diagnostic markers.

**Figure 1:**
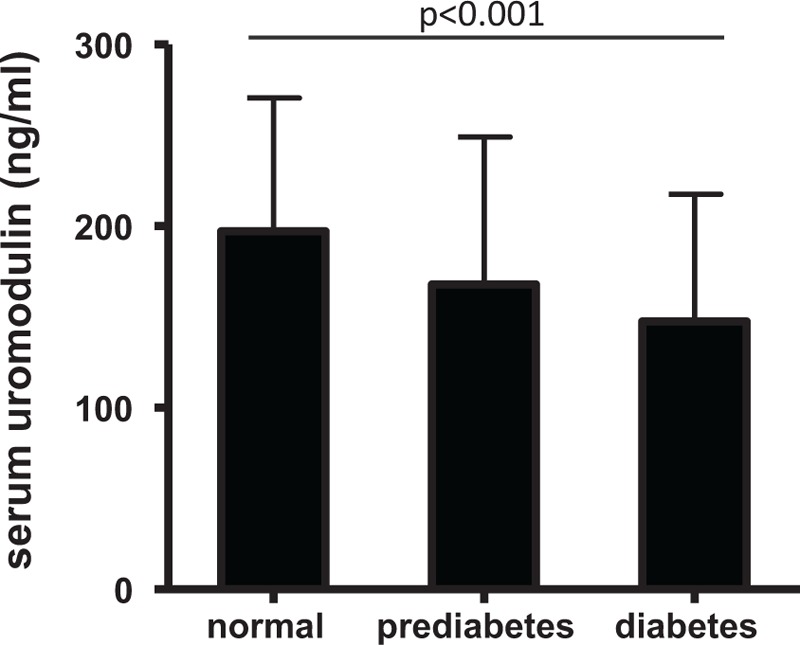
Serum uromodulin in nondiabetic, prediabetic, and diabetic patients uromodulin concentration in patient subgroups with unimpaired glucose metabolism (normal), impaired metabolism (prediabetes), and established T2DM (diabetes) according to ADA definition. The *P*-value is given for trend. ADA = American Diabetes Association, T2DM = type 2 diabetes mellitus.

With respect to the ADA definition for diabetes including FPG, OGTT, HbA1c, and antidiabetic treatment 146 patients had type 2 diabetes (27.6%) and we revealed significantly lower uromodulin concentrations in patients with type 2 diabetes than among nondiabetic patients (147.7 ± 69.9 vs 171.4 ± 78.9 ng/mL; *P* = 0.001). Moreover, if only nondiabetic subjects were taken into account, we found that patients who have been assigned to prediabetes according to ADA definition (FPG: 100–125 mg/dL; OGTT: 140–199 mg/dL, and HbA1c: 5.7%–6.4%) had a concentration of 166.5 ± 79.4 ng uromodulin per mL serum, whereas those without prediabetes had 180.3 ± 75.5 ng uromodulin per mL serum (*P* = 0.028). A comparison between these 3 groups is given in Fig. 1. In addition, we then separated the study cohort with respect to ADA-defined thresholds for FPG, OGTT glucose, and HbA1c (Table 3). Serum uromodulin concentration was highest in patients with normal FPG, normal OGTT glucose, and normal levels of HbA1c. It was decreased in patients with impaired fasting glucose, impaired glucose tolerance (IGT), and elevated HbA1c, and lowest in patients, in which FPG, OGTT glucose, or HbA1c were above the diabetes-defining threshold. This trend was found significant in all 3 groups (p_trend_ < 0.001, <0.001, and =0.008, respectively). In accordance, if separating the study cohort according to tertiles of eAG/FPG ratio (0.47–1.14, 1.14–1.26, and 1.26–4.42), uromodulin concentrations were 148.7 ± 62.0, 171.9 ± 87.0, and 173.6 ± 78.5 ng/mL (p_trend_ = 0.005).

**Table 3:**
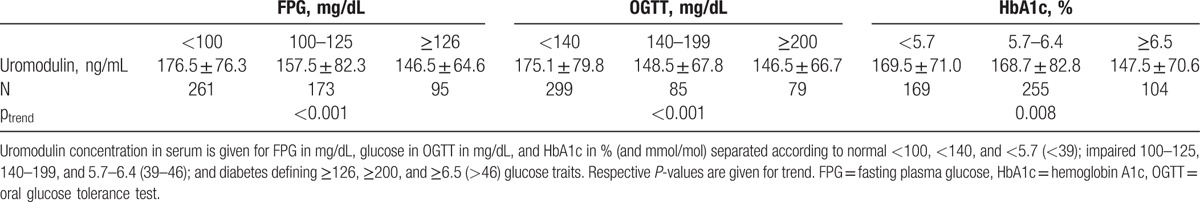
Serum uromodulin concentration in categories for diabetes classification.

In order to assess a possible impact of antidiabetic drugs, we compared subjects diagnosed to have diabetes, but we did not see a significant difference between those who were under antidiabetic treatment (149.9 ± 75.9 ng/mL, n = 76) and those not taking antidiabetic drugs (145.3 ± 63.2 ng/mL, n = 70, *P* = 0.754). In addition, if separating patients with respect to the median of uromodulin concentration, patients with low uromodulin concentration were significantly more often affected by T2DM, than patients with high uromodulin concentration (*P* = 0.006).

In line with univariate results, ANCOVA adjusting for age, gender, BMI, hypertension, smoking, CAD, and even estimated GFR revealed that T2DM is significantly and independently associated with uromodulin concentration in serum (F = 5.5; *P* = 0.020). Additional adjustment models including ACR and CRP are given in Table 4 further approving this significant association. A similar result was seen if, instead of T2DM, the beta cell function given as eAG/FPG ratio was used in ANCOVA. It revealed a significant and independent association with uromodulin with age, gender, BMI, hypertension, smoking, CAD, and even estimated GFR as covariates (F = 5.9; *P* = 0.015). Applying the same adjustment model for the subgroup of nondiabetic patients, prediabetes, as defined above, barely escaped statistical significance (F = 3.6; *P* = 0.059).

**Table 4:**
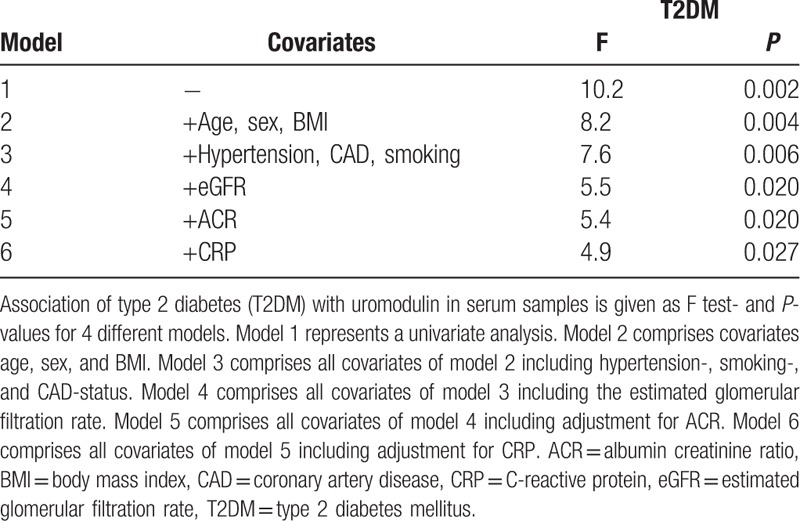
Analysis of covariance for the association of T2DM with serum uromodulin.

Prospectively, regarding the incidence of diabetes we saw that 146 patients with T2DM at baseline had a uromodulin serum concentration of 147.7 ± 69.9 ng/mL. Twenty-one of our patients who did not have T2DM at baseline but developed T2DM during 4 year FU had 164.0 ± 66.8 ng/mL, and 241 patients who did not have T2DM at baseline and did not develop T2DM during 4 year FU period had 179.9 ± 82.2 ng/mL uromodulin at baseline (p_trend_ < 0.001). Similar results were seen with respect to the incidence of prediabetes in patients without diabetes at baseline. Serum uromodulin was lowest in patients who were already characterized at baseline to have prediabetes (168.0 ± 81.2 ng/mL), higher in patients who developed prediabetes during FU (172.8 ± 66.3 ng/mL), and highest in those who were not characterized to have prediabetes at baseline and did not develop prediabetes during FU (188.2 ± 74.0 ng/mL; p_trend_ = 0.011).

## 4 Discussion

In the present study, we demonstrate for the first time that serum uromodulin is significantly associated with glucose metabolism and that patients with impaired glucose metabolism, prediabetes, or diabetes have significantly lower concentrations of uromodulin in serum than unimpaired or nondiabetic subjects.

Likewise, patients with low uromodulin serum levels were more often affected by T2DM than those with high levels, and T2DM has been demonstrated to be an independent determinant of serum uromodulin in view of basic confounders including CRP and even with respect to kidney function in terms of estimated GFR, ACR, and hypertension.

Decreased urine excretion of uromodulin has been described earlier in children with type 1 diabetes.^[10]^ Our data for uromodulin in serum are comparable to the range previously reported for healthy individuals with serum uromodulin between 70 and 540 ng/mL by Dawnay and Cattell,^[30]^ and between 45 and 490 ng/mL by Risch et al^[13]^ respectively, and slightly higher as reported for chronic kidney disease patients by Steubl et al^[14]^ with plasma uromodulin between 3 and 312 ng/mL. Moreover, our data also support previous study results for urinary uromodulin suggesting an association between diabetes and decreased uromodulin: a first link between decreased urine uromodulin concentration and diabetes has been reported in 1987.^[9]^ Some years later, these data could be confirmed in urinary samples of postmenopausal women.^[31]^ In immunogold labeling experiments of kidney tissue, samples from patients with diabetes and also with dysfunctional kidneys have been shown to contain less uromodulin protein than control samples.^[32]^ In contrast, Torffvit et al,^[11]^ using an enzyme linked immunoassay, did not find a significant difference between 58 type 1 diabetes patients and 76 control subjects.

In summary, our data are therefore in line with the majority of data for urinary uromodulin. Given that analogy of results between urinary and serum uromodulin, we believe that our data may corroborate the hypothesis of a link between urinary and serum uromodulin concentration, which however needs fortification in future studies.^[3,33,34]^ Moreover, this is the first study demonstrating significantly lower uromodulin concentrations in serum of diabetic patients than in nondiabetic patients. In accordance, serum uromodulin in our patients was inversely correlated with blood glucose parameters FPG, 2 hour OGTT glucose, and HbA1c. Thus, a very consistent picture arises, linking diabetes and low serum uromodulin.

For HbA1c, our data are in line with data of Torffvit et al.^[11]^ They have reported a correlation between urinary uromodulin and HbA1c for type 1 diabetic patients, but did not find a correlation with age, blood pressure, and antihypertensive treatment.^[11]^

In this context, the difference between eAG, which directly correlates to HbA1c, and FPG has been previously demonstrated to be significantly different between subgroups in diabetic and nondiabetic subjects, and it has been suggested to be associated with glycemic control.^[35]^ In addition, the eAG/FPG ratio has been previously used to assess β-cell function.^[25]^ In our study, the eAG-FPG difference as well as the eAG/FPG ratio were both correlated with uromodulin and this was also true for the correlation with HOMA-β and ISI. This might suggest that uromodulin could play a role for glycemic control as well as for β-cell function. On the other hand, we did not find a correlation between uromodulin and HOMA-IR or insulin level. With respect to antidiabetic treatment in patients with kidney disease, as discussed for metformin,^[36]^ it appears important that no significant difference was seen in our study between diabetic patients who were under antidiabetic treatment and those who were not. Thus, further studies appear necessary to elucidate uromodulin's role.

Due to the prospective character of our study, we observed in our study population that low uromodulin concentration was associated with the development of T2DM, and even of prediabetes.

There are also some open question and limitations to be mentioned. First, neither the detailed function nor the regulation of uromodulin or the source and detailed transport in case of serum uromodulin are yet known. Second, uromodulin levels may decrease very early even in chronic kidney disease stage 1 where creatinine and eGFR are still within the normal range.^[14]^ In the context of renal involvement of diabetes, we observed higher ACR but lower eGFR in our coronary patients, and we adjusted for eGFR and ACR with respect to the association between diabetes and uromodulin. However, we do not know the time when patients’ kidney function started to get worse, if at all. Thus, we cannot exclude a preexisting renal involvement in our patients. Third, some histopathological alterations including glomerular, tubular, and interstitial changes can precede clinical and traditional laboratory criteria of early renal involvement in T2DM. In that context, the role of tubular injury and excretion of tubular proteins in early stages of diabetic nephropathy have been repeatedly emphasized.^[37–44]^ Moreover, uromodulin is expressed primarily in the thick ascending limb, but expression elsewhere,^[45,46]^ even regarded to be negligible,^[47]^ may play a role for measurement in serum samples as concentrations of uromodulin in serum has been mentioned previously to be lower by factor about 1000 compared to urine.^[48]^ This can be roughly estimated from about 20 mg uromodulin per gram creatinine in urine and about 15 ng uromodulin per mL serum seen in control subjects by Prajczer et al^[49]^ assuming a daily mean secretion of about 1 g creatinine with about 1500 mL urine. However, their findings on patients with renal failure showing elevated serum uromodulin^[49]^ contradicts to all recent observations as mentioned above. Fourth, the study was confined to patients with a mean age of 65 years undergoing coronary angiography for the evaluation of CAD who are therefore a selected group of coronary patients and thus do not reflect the general population. Nevertheless, these subjects deserve particular clinical interest as they represent a patient cohort under high risk. As opposed to these limitations, a particular strength of our study is its prospective character and the precise characterization of participants.

## 5 Conclusion

This study demonstrates for the first time that uromodulin in serum is associated with impaired glucose metabolism. Thus, serum uromodulin is not only a promising biomarker for identifying even early stages of kidney disease; it also allows the detection of diabetes and even prediabetes. Apart from its value in kidney patients, we hope that uromodulin, according to the present study data, might become a part of routine diagnostics in high-risk patients in view of metabolic dysregulation and diabetes.
